# A Theory for the Origin of Human Menopause

**DOI:** 10.3389/fgene.2016.00222

**Published:** 2017-01-06

**Authors:** Mike Takahashi, Rama S. Singh, John Stone

**Affiliations:** Department of Biology, Origins Institute, McMaster University, HamiltonON, Canada

**Keywords:** adaptation, exaptation, fertility, genetic theory, lifespan, mating behavior, neutral evolution, senescence

## Abstract

A complete and compelling evolutionary explanation for the origin of human menopause is wanting. Menopause onset is defined clinically as the final menses, confirmed after 1 year without menstruation. The theory proposed herein explains at multiple levels – ultimately genetic but involving (1) behavioral, (2) life history, and (3) social changes – the origin and evolution of menopause in women. Individuals in Lower Paleolithic human populations were characterized by short lifespans with diminished late-age survival and fertility, similar to contemporary chimpanzees, and thence were subject to three changes. (1) A mating behavior change was established in which only young women reproduced, thereby rendering as effectively neutral female-specific late-onset fertility-diminishing mutations, which accumulated subsequently. (2) A lifespan increase was manifested adaptively, revealing the reproductive senescence phenotype encoded in late-onset fertility-diminishing mutation genotypes, which, heretofore, had been unexpressed in the shorter lifespan. (3) A social interaction change emerged exaptively, when older non-reproductive women exclusively started assisting in rearing grandchildren rather than giving birth to and caring for their own children, ultimately leading to menstrual cycle cessation. The changes associate in a one-to-one manner with existing, non-mutually exclusive hypotheses for the origin of human menopause. Evidence for each hypothesis and its associated change having occurred are reviewed, and the hypotheses are combined in a synthetic theory for the origin of human menopause. The new theory simultaneously addresses the main theoretical problem with each hypothesis and yields predictions for future testing.

## Menopause – Definition, Description in Relation to Fertility, and Consideration As A Life History Phenotypic Trait with Genotypic Ultimate Causes

Human menopause is defined as menstrual cycle cessation and usually is recognized 1 year after the final menses (Col et al., 2009). Follicle number starts to decline very early in female ontogeny (Gosden and Faddy, 1998). Germ cell numbers peak at approximately 3 × 10^5^ (Hamish et al., 2010) to 7 × 10^6^ (Gosden and Faddy, 1998), approximately 5 months into the gestation period, decreasing to approximately 3.5 × 10^4^–2.5 × 10^6^ by birth (Gosden and Faddy, 1998; Hamish et al., 2010). Artesian is the primary cause for follicular loss, even after puberty (Gosden and Faddy, 1998), ovulation accounting for little (Gosden and Faddy, 1998). Follicles undergo programmed atresia throughout female lifespan, with onset occurring comparatively early and rate increasing toward female reproductive cessation, typically doubling by approximately 37 years (Gosden and Faddy, 1998). Follicle depletion thus ultimately and technically is the cause for menopause (Armstrong, 2001; Col et al., 2009). Oocyte quantity also decreases with age, likely due to increase in defects (Armstrong, 2001; Chichester and Ciranni, 2011). Oocytes develop during folliculogenesis and become mature by the tertiary phase (0.2–20 mm; Armstrong, 2001). Oocytes containing unrepaired meiotic or induced DNA mutations are culled through checkpoint genetic pathways (Titus et al., 2013; Bolcun-Filas et al., 2014).

Declining fertility can present before total depletion (Armstrong, 2001; Chichester and Ciranni, 2011), gradually between ages 35 and 40 years, followed by a rapid decline (Armstrong, 2001). The cause for reduced fertility with age likely results from defects in oocytes (Armstrong, 2001). Although the mechanisms responsible are understood poorly (Armstrong, 2001), genetic factors are known or suspected to be involved. Traits like premature menopause onset have been documented as familial characteristics (Qian et al., 2012), with heritability estimated at 30–85% (Wicks et al., 2004). Research on patients with premature ovarian failure has implicated candidate genes (e.g., GDF9, BMP15 and FOXL2; Jagarlamudi et al., 2010), and research with mutant mouse models (e.g., GDF9-/-, FSHR-/-, and ER-/-) has hinted at genes (Hamatani et al., 2004) and molecules (Willer et al., 2006; Lakhal et al., 2010) that might be involved. Studies on SNPs and linkage (Welt et al., 2004) have revealed chromosomal regions that associate with reproductive aging (Petros et al., 2004), including apoptosis pathways (Hsu and Hsueh, 2000). Morphological and meiotic abnormalities additionally might contribute to reduced fertility with increased age (Armstrong, 2001).

Hormones also play a role. Hypothalamic-pituitary abnormalities, ovarian endocrine deficiencies, and impaired oviduct functioning can cause fertilization failure and decreased endometrial receptivity (Armstrong, 2001). Follicle-stimulating hormone and luteinizing hormone levels increase throughout menopause (Col et al., 2009), while estrogen levels decrease (Kato et al., 1998; Chichester and Ciranni, 2011). Evidence for endometrial function affecting female fertility is consistent with the observation that aging women can increase fertility with hormone therapy (Armstrong, 2001).

The age at which menstruation stops in Caucasian women in industrialized countries is 50.1–51.5 (McKinlay, 1996; median 51.3; Col et al., 2009), typically preceded by irregular menses for 4 years (Col et al., 2009). Menopause thus constitutes an evolved stage in human female life history.

The preceding data reveal that, as with other life history traits, menopause manifests at a variety of levels (e.g., individual, cell, and molecule). Its ultimate cause, however, resides at the genetic level. The new theory proposed herein suggests that menopause arose from three changes, with effects and causes that also reside ultimately at the genetic level.

## Changes and Hypotheses

### Change 1: Mate-Choice Hypothesis

A recently published, novel population genetic hypothesis states that a change in mating behavior – involving only young adult females and adult males – provides a means for evolving a menopause phenotype (Morton et al., 2013). Such a change in reproductive dynamics within populations would relax selection on older females. Effects from female-specific late-onset fertility-diminishing mutations then would be rendered effectively neutral, and mutant alleles would accumulate over time, eventually fixing and leading to reproductive senescence (i.e., diminished fertility at older ages) and, ultimately, menstruation cessation. Reproductive senescence was considered implicitly in the hypothesis as a prelude to an ensuing menopause, caused ultimately by the accumulated mutant alleles. Real-world counterparts for those alleles might be the aforementioned genetic factors associated with menopause (described in the previous section). We hereafter refer to diminished fertility followed by the final menses collectively as a ‘menopause phenotype,’ which may be defined generally and formally from a life history evolution perspective as a lengthy adulthood within which the reproductive system has senesced and menstrual cycles have ceased for a proportionately large fraction (Peccei, 2001; Levitis and Lackey, 2011).

The new hypothesis was introduced in association with a computational model, and computer simulation was used to test whether predicted survival and fertility curves could be produced under particular scenarios. Some scenarios involved populations in which individuals initially survived into old age, subject to effects from late-onset survival-diminishing mutations; population size was constant, with deaths compensated for by births from pseudorandomly chosen mating pairs (in contrast to a subsequent interpretation; Hawkes and Coxworth, 2013). If female fertility intrinsically remained diminished at mid life, then standard, real-world survival and fertility curves were produced whether reproduction involved all or only young adults. Purifying selection (operating on male fertility) against late-onset, survival-diminishing mutations prevented them from accumulating in females, and mutation-selection balance prevented fixation. This confirmed prediction corroborated a previously demonstrated, fundamental role that males might have played in menopause phenotype evolution in humans (Tuljapurkar et al., 2007), enabling females to live beyond their reproductive years, overcoming the proverbial ‘wall of death’ expected on the basis of classic theory (Cole, 1954; Hamilton, 1966).

Another potentially fundamental role was demonstrated by including in additional scenarios effects from gender-specific late-onset fertility-diminishing mutations (Morton et al., 2013). If female fertility initially extended into old age, then standard, real-world survival and fertility curves were produced when reproduction involved only young adult females and adult males. Two cases, therefore, younger females out-competing older females for access to mates (e.g., Cant and Johnstone, 2008) or, equivalently, male preference for younger females (e.g., Sugiyama, 2005), ultimately could produce a menopause phenotype. Requiring only a switch in mating behavior to produce a menopause phenotype rendered the ‘mate choice hypothesis’ (Morton et al., 2013) parsimonious and, from a scientific hypothesis perspective, boldly falsifiable (Popper, 1987; perhaps too bold for consideration on its own scientific merit; Clancy, 2013; Saini, 2014).

The mate choice hypothesis (Morton et al., 2013) is sufficient to explain generally how a genotype encoding a menopause phenotype can originate and evolve in a population. But sufficiency is neither necessary nor necessarily convincing for explanation with any natural phenomenon. Among the main theoretical problems facing the mate choice hypothesis and accompanying computational model as they apply to humans, the most challenging, previously unaddressed problem involves when in human history longevity became extended. The computer simulations (Morton et al., 2013) suggested that tens- or perhaps hundreds-of thousands of years would be required for female-specific late-onset fertility-diminishing mutations to accumulate and ultimately produce a menopause phenotype. Human populations might have lived long lives for an insufficient time period to have accommodated such an accumulation. This ‘increased longevity timing’ challenge to the mate choice hypothesis could be addressed by altering in the computational model locus number and magnitudes for deleterious effects associated with female-specific late-onset fertility-diminishing mutations. But adopting that approach would reduce explaining the origin of the menopause phenotype in our species merely to searches for appropriate, explanatory regions in that particular parameter space. Computational models provide practical means for describing reality at particular levels and under specified assumptions, the population genetic computational model used in conjunction with the novel mate choice hypothesis as only and explicitly a means for testing whether a menopause phenotype could be produced through accumulating late-onset fertility-diminishing mutations that had been rendered effectively neutral by a change in mating behavior. The increased longevity timing challenge instead ultimately is addressed herein by synthesizing in the genetic theory the ‘lifespan artifact hypothesis’ (see subsequent section The Theory). That mate preference and behavior shifts have occurred among primates is documented in the next section.

#### Mating Preferences and Behavior Shifts

Mating preferences vary among primate species (**Table 1**). Male chimpanzees prefer mating with older rather than younger females, in contrast to humans (Muller et al., 2006). Three behaviors that are absent in chimpanzees but present in humans might explain this difference. Humans form long-term mate bonds, have an extended post-reproductive period, and offer paternal care (Muller et al., 2006). During the least-fertile phase in female chimpanzee menstrual cycles, males tend to prefer grooming females who are in their peak reproductive years (14–25 years; Proctor et al., 2011); during the most-fertile phase, males tend to prefer grooming older females who previously had produced offspring (Proctor et al., 2011). Males also routinely groom females who are in their peak reproductive years (ages 14–25) and already have produced offspring (rather than focusing all grooming efforts on older and most-reproductive females) and thereby may be demonstrating a trade-off in reproduction, as these younger females might provide better mating opportunities in the future (Proctor et al., 2011).

**Table 1:** Mating preferences among primate species (female age preferred by males for mating across several primate groups).

Primate	Female age preferred by males
Humans	Women 30–40 (Anderson, 1986)
	Women <25 (Buss, 1989)
	Women younger than themselves (Kenrick and Keefe, 1992)
Chimpanzee	Older for mating, younger for socializing (Anderson, 1986; Muller et al., 2006; Proctor et al., 2011)
Gorilla	Adult (over age of 8; Anderson, 1986; Watts, 1991; Robbins, 1999)
Bornean orangutan	Preference for older parous females (Anderson, 1986)
Olive baboons	Males prefer to mate with older females, adolescent females mate with adolescent males (Anderson, 1986)


*In some studies, only relative ages could be inferred from general physical characteristics.*

Mating behavior shifts related to estrus cycles have been observed among primate species. In rhesus macaques, males engage in mate guarding behavior when females are at peak fertility (Dubuc et al., 2012). This behavior involves aggression toward other males, protecting females (Dubuc et al., 2012). Females appear to mask partially their fertility from males, which allows females to exert more control in their reproduction, selecting preferred males at peak fertility (Dubuc et al., 2012). In modern humans in industrialized societies, women at peak fertility in their menstrual cycle are more attracted to charismatic, dominant men (Durante et al., 2012). Men often over-estimate preference by women for male-dominated relationships (Kruger and Fitzgerald, 2011). Men who use short-term mating strategies are more likely to be considered physically attractive and socially dominant but lack traits associated with long-term partnership and fatherhood (Kruger and Fitzgerald, 2011; Durante et al., 2012). Men who use long-term mating strategies tend to be less dominant, charismatic, and physically attractive but possess traits associated with long-term partnership and parenting (Kruger and Fitzgerald, 2011; Durante et al., 2012). Women exhibit shifts in preference from long- to short-term mates as women enter fertile phases in their menstrual cycles, to capitalize on ‘good genes’ and the personal characteristics and investing behavior characterizing long-term males (Durante et al., 2012). Women entertainers at the fertile phase in their menstrual cycle earn significantly more money in tips than do counterparts who are either on contraceptive pills or outside their fertile phase (Miller et al., 2008). This implies that mating decisions made by men can result from immediate context and behavior toward individual women can change during menstrual cycles.

A lesser known mating behavior change involves partiable paternity, wherein multiple men are considered as fathering individual offspring. This behavior has been documented in some South American societies (Walker et al., 2010). Partiable paternity is hypothesized to enable women to secure good genes and resources from available men (Walker et al., 2010); it also provides some men with access to mating opportunities that they otherwise never would have (Walker et al., 2010). This mating behavior also may be beneficial for creating more diverse social alliances (Walker et al., 2010).

At some point in homininan evolution, a major change occurred, wherein males shifted efforts from mating to also engaging in rearing offspring and began to benefit from providing care to offspring (Geary, 2000). Higher paternal investment levels are correlated with better quality offspring, in social status and survivorship terms, among other traits (Geary, 2000).

### Change 2: Lifespan Artifact Hypothesis

The lifespan artifact hypothesis states that increased longevity could have provided a means for the menopause phenotype to have evolved in humans (Washburn, 1981; Weiss, 1981; Gosden and Telfer, 1987; O’Rourke and Ellison, 1993; Austad, 1994; Leidy, 1999; Wood et al., 1999; Marlowe, 2000; Peccei, 2001; reviewed in Morton et al., 2013). Modern humans live longer than did our ancestors, and females outlive their innate reproductive capacities. Archeological analyses yield estimates that premodern human lifespans extended to only 50 or 55 years (Hill and Hurtado, 1991), approximately 15–30 years shorter than estimates for individuals in contemporary industrialized societies (Gavrilov and Gavrilova, 2002). The related statistic ‘life expectancy at birth’ has been shown to have increased over time (**Table 2**).

**Table 2:** Life expectancy at birth for time periods in human history.

Time period	Estimates for life expectancy at birth (years)
Neolithic (12200-4000 a)	Early 20s (Acsádi and Nemeskéri, 1970) 25 (Bocquet-Appel and Bar-Yosef, 2008)
Copper age (7000-3200 a)	Late 20s (Acsádi and Nemeskéri, 1970)
Roman Egypt (2000 a)	Mid-late 20s (Acsádi and Nemeskéri, 1970)
Roman Empire	Slave: 17.2 (male); 17.9 (female) professionals: 40.3 (male); 23.1 (female; Acsádi and Nemeskéri, 1970)
Middle ages (1500-500 a)	27–29 (Gavrilov and Gavrilova, 2002)
Developed countries (current)	70–80 (Gavrilov and Gavrilova, 2002)


*Life expectancy can be calculated by integrating survival curves from age 0 to maximum lifespan.*

Most explanations for increased longevity involve extrinsic factors, such as avoiding predation, improving living conditions and health, and industrialization. Explanations involving intrinsic factors include genetic components, whether indirectly (e.g., through energy considerations; Austad and Fisher, 1991) or directly (such as telomere degradation; Hayflick and Moorhead, 1961; Hayflick, 1965; Donate and Blasco, 2011; Shay and Wright, 2011). Telomere and telomerase variation in particular have been cited as factors in extending human lifespan (Atzmon et al., 2010). Genes associated with lifespan, involving pleiotropic trade-offs between early reproduction and death, have been identified in non-human animals (e.g., *methuselah* in fruit flies; Lin et al., 1998; *age-1* in nematodes; Walker et al., 2000); and longevity has been demonstrated to be a trait upon which selection can operate (Luckinbill and Clare, 1985). Whether and how these genetic factors affect human lifespan is unknown.

The lifespan artifact hypothesis explanation implies that the menopause phenotype arose after modern humans had decreased extrinsic causes for mortality and predicts that the onset age for the menopause phenotype has increased over time. Analyses conducted on data that were collected to test this hypothesis have yielded equivocal results (Howell, 1982; Melacon, 1982; Buikstra and Konigsberg, 1985; Early and Peters, 1990: Hill and Hurtado, 1991; Austad, 1994). Individuals in contemporary hunter-gatherer societies (e.g., !Kung in present-day Namibia, Botswana and in Angola; Yanomami in present-day Venezuela and Brazil; and Aché in present-day Paraguay) live beyond 70 years (Hill and Hurtado, 1991), for instance. The lifespan artifact hypothesis on its own has as its most challenging theoretical problem addressing why only fertility in men was extended concomitantly with increased longevity.

### Change 3: Grandmother Hypothesis

The grandmother hypothesis states that inclusive fitness benefits accrued by older women through assisting in rearing grandchildren rather than giving birth to and caring for their own children could have provided a means for the menopause phenotype to have evolved in humans (Williams, 1957; Hamilton, 1966; Trivers, 1972; Alexander, 1974; Gaulin, 1980; Hawkes et al., 1989, 1998; Hill and Hurtado, 1991; Austad, 1994; Sean et al., 2000; Peccei, 2001; Shanley and Kirkwood, 2001; Jamison et al., 2002; Kaplan and Robson, 2002; Hawkes, 2003; Lee, 2003; Hrdy, 2005; Voland et al., 2005; Shanley et al., 2007; Kim et al., 2012; Hawkes and Coxworth, 2013; reviewed in Morton et al., 2013). Grandparents constitute among the greatest examples for kin selection in contemporary human populations. Sociological analyses have shown that humans tend to behave in a manner consistent with kin selection theory (Hamilton, 1964), favoring relatives over non-relatives in sharing food (Betzig and Turke, 1986; Ziker and Schnegg, 2005), providing resources (Smith et al., 1987), and provisioning care (Davis and Daly, 1997). Definitions and descriptions for the grandmother hypothesis have varied over time and across authors (**Table 3**).

**Table 3:** Definitions and descriptions for the grandmother hypothesis over time and authors.

Source	Definitions/function of the grandmother hypothesis
Shanley and Kirkwood (2001, p. 282)	“…menopause enhances fitness by producing post-reproductive grandmothers who can assist their adult offspring by sharing in the burden of provisioning and protecting their grandchildren.”

Hawkes (2003, p. 386)	“[mother-offspring provisioning enabled by grandmothers] creates a novel fitness opportunity for older females whose own fertility is declining. If the older females help feed their just-weaned grandchildren, the mothers of those weanlings can have shorter interbirth intervals without reductions in offspring survivorship. The more vigorous elders who have no nursing infants of their own will thus raise their daughters’ reproductive success.”

Hawkes et al. (1998, p. 1336)	“Long post-menopausal lifespans distinguish humans from all other primates. This pattern may have evolved with mother-child food sharing, a practice that allowed aging females to enhance their daughters’ fertility, thereby increasing selection against senescence.”

Peccei (2001, p. 434)	“In the old grandmother hypothesis, menopause is an adaptation facilitating grandmothering; it is about stopping early in order to create a post-reproductive lifespan. In the new grandmother hypothesis, grandmothering is an adaptation facilitating increased longevity, and menopause is a byproduct.”


Most explanations for older women halting reproduction implicitly involve kin selection (whether stated explicitly). Older women historically risked danger due to senescing physiological systems and mortality from predation in surviving to produce next offspring as well as increased demands for resources as their daughters matured to reproductive age to produce their own offspring. Foregoing future, risky reproduction to help rear kin suggests that non-reproductive adult human females historically should have been industrious in provisioning for grandchildren. Older women in contemporary hunter gatherer societies, indeed, are among the most productive in foraging and sharing food, consistent with kin selection theory (Hawkes et al., 1989). Whether inclusive fitness gains are sufficient to explain reproduction cessation remains contentious.

The grandmother hypothesis explanation implies that the menopause phenotype is adaptive and predicts that older women historically overcame the decrease in gene-pool contributions inherent in the twofold reduction in genetic relatedness typically observed over generations. Analyses conducted on data that were collected to test this hypothesis have yielded equivocal results (Hill and Hurtado, 1991, 1996; Peccei, 1995, 2001; Alvarez, 2000; Lahdenperä et al., 2004; Kuhle, 2007; Madrigal and Meléndez-Obando, 2008; Hawkes et al., 2011; Kachel et al., 2011). Grandmothers in contemporary hunter-gatherer societies (e.g., Aché) typically gain through kin selection only approximately 5% an additional offspring (Hawkes et al., 1998). Researchers also have tested whether menopause, itself, is adaptive and found that, in premodern European (e.g., Finnish) societies, grandmothers gained 2 additional grandchildren per decade past age 50 years (Lahdenperä et al., 2004); in other premodern (e.g., Costa Rican) societies, in contrast, longer lifespan was associated with fewer grandchildren (Madrigal and Meléndez-Obando, 2008). No research has demonstrated genetic accounting consistent with inclusive fitness gains expected through kin selection to compensate for lost individual fitness. The grandmother hypothesis on its own has as its most challenging theoretical problem addressing this steep selective gradient.

A recently published computational model and associated computer simulation study showed that “grandmothering” – caring for any weaned dependent by post-fertile females rather than giving birth to and caring for their own children – could produce an extended lifespan phenotype (Hawkes and Smith, 2010; Kim et al., 2012; Hawkes and Coxworth, 2013). This intriguing result implies that grandmothering evolved before reproductive senescence and extended longevity. Care from non-reproductive women and associated effects were an important factor in human evolution and continue to be in modern human societies. But whence grandmothers (i.e., whether biological and benefiting from kin selection; Williams, 1957; Hamilton, 1966; Trivers, 1972; Alexander, 1974; Gaulin, 1980; Hawkes et al., 1989, 1998; Hill and Hurtado, 1991; Austad, 1994; Sean et al., 2000; Peccei, 2001; Shanley and Kirkwood, 2001; Jamison et al., 2002; Kaplan and Robson, 2002; Hawkes, 2003; Lee, 2003; Hrdy, 2005; Voland et al., 2005; Shanley et al., 2007; Kim et al., 2012) or functional and presumably acting in a reciprocally altruistic manner (Kim et al., 2012; Hawkes and Coxworth, 2013)? Explaining the origin of human grandmothers is challenging, especially given the twofold-increased selective gradient?

Speculations that preference for younger women arose only after reproductive senescence had arisen (Cohen et al., 2013; Levitis and Cohen, 2013; Stone, 2013) similarly lack an initiating cause for diminished fertility (i.e., grandmothers). We prefer involving the mate choice hypothesis in menopause-origin scenarios because it constitutes an explanation for how grandmothers originated, and we propose alternatively that genuine grandmothering evolved later, as a consequence from accumulated mutations and extended longevity, reinforcing their associated effects. Genuine grandmothering, from this perspective, constitutes an elaboration on parental care (specifically maternal), which had evolved earlier. Whether genuine grandmothering evolved prior to paternal care (Geary, 2000) becomes an interesting topic for future research. Relationships between grandmothers and kin interestingly appear to have an effect on offspring survivorship: maternal grandmothers offer benefits to grandchildren whereas paternal grandmothers increase risk for child mortality (Jamison et al., 2002; Voland and Beise, 2002). Effects associated with grandmothers, therefore, are non-universal.

## The Theory

**FIGURE 1:**
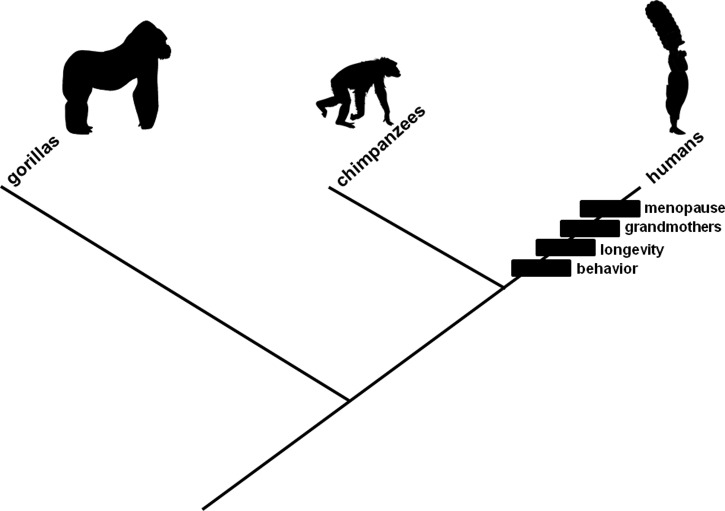
**Evolutionary branching diagram presented as a phylogenetic tree depicting relationships among gorillas, chimpanzees, and humans.** The rectangles represent changes, characteristics that evolved in the human lineage: mating involving young adult women (“behavior”), increased lifespan (“longevity”), care for grandchildren by older non-reproductive females (“grandmothers”), and a proportionately long time in adulthood during which individuals are non-reproductive (“menopause”). Hypotheses (cited and described in the text) exist to explain each change. Relationships among menopause and the other three characteristics as well as their associated hypotheses are contentious. The characteristics are presented in the order in which they are described (and theorized to have evolved) in the text.

Contemporary human populations are characterized by mating behaviors favoring young adult females (whether through intergenerational competition or male preference), long lifespans, and non-reproductive older females, but whether these characteristics arose, respectively, in scenarios associated with the mate choice, lifespan artifact, or grandmother hypothesis remains controversial. Contemporary human populations also are characterized by menopause, and the relationships among this characteristic and the other three characteristics as well as their associated hypotheses are contentious (**Figure 1**). We provide herein, in a narrative framework, a theory for the origin and evolution of the human menopause phenotype. The theory involves as a sufficient, mechanistic, neutral genetic originating explanation, the mating behavior change described in the mate choice hypothesis. This is combined with a necessary, adaptive, initiating explanation, the lifespan increase described in the lifespan artifact hypothesis. These two explanations, together, account for the origin of reproductive senescence. They are accompanied by a contributory, exaptive, emerging explanation, a social interaction change in which older non-reproductive women exclusively started assisting in rearing grand-offspring rather than giving birth to and caring for additional offspring, described in the grandmother hypothesis; this accounts genetically for reproductive cessation among older adult females emerging in an evolving population, ultimately leading to the menopause phenotype that is observed in modern human societies.

The theory for the origin of human menopause is formulated by involving the mate choice hypothesis with the lifespan artifact hypothesis and grandmother hypothesis. Human populations initially were characterized by a relatively short individual lifespan with diminished survival and fertility toward the end, as in contemporary chimpanzee populations. A change in mating behavior was established in which only young women mated with men. The change in mating behavior rendered as effectively neutral late-onset fertility-diminishing mutations in women. Mutant alleles accumulated in women. An increase in longevity was manifested for adaptive reasons such as decreased mortality or increased fitness resulting from prolonged, reproductively active adulthoods. The increased longevity revealed reproductive senescence (i.e., in women, encoded in late-onset fertility-diminishing mutation genotypes), which, erstwhile, had been unexpressed in the shorter, ancestral lifespan. Expressing this phenotype provided an opportunity for kin selection to operate exaptively, when older non-reproductive women (i.e., the first grandmothers) exclusively assisted in rearing their offsprings’ offspring rather than giving birth to and caring for new ones, during post-fertile adulthood, ultimately leading to the menopause phenotype associated with contemporary human populations.

A crucial detail in this narrative concerns relative timing for the hypothesized change in mating behavior and documented increase in lifespan. The change in mating behavior logically could have preceded, coincided with, or followed the increase in lifespan. The latter scenario was adopted previously (Morton et al., 2013) to demonstrate emphatically how mutation accumulation could lead to reproductive senescence. Emphasis was achieved by requiring the change in mating behavior to diminish fertility from a hypothetical, biologically unreal, maximally extended condition rather than a parsimonious, ancestral (but mysteriously intrinsically determined) decline in fertility at a predetermined age, which would have been uninteresting computationally. Evidence exists that mating behavior involving young adult women is favored in our lineage, whether through male preference (Symons, 1979; Casterline et al., 1986; Buss, 1989; Jones, 1996; Sugiyama, 2005) or intergenerational conflict (Cant and Johnstone, 2008; Lahdenperä et al., 2012), an autapomorphic character state among the Homininae (**Figure 1**, “behavior”). Researchers have documented that human populations evolved dramatic increases in lifespan in the Upper Paleolithic (Hawkes et al., 1998; O’Connell et al., 1999; Hawkes, 2003; Caspari and Lee, 2004; Hawkes and O’Connell, 2005; Kachel et al., 2011), also an autapomorphic character state among the Homininae (**Figure 1**, “longevity”). This documentation provides up to approximately 50000 years for female-specific late-onset fertility-diminishing mutations to have accumulated, allowing precedence or coincidence as plausible alternatives. Non-reproductive older females subsequently could recoup evolutionarily fitness lost in unrealised potential matings (e.g., Hawkes, 2003) by caring for grandchildren (**Figure 1**, “grandmothers”).

The theory retains the salient point from the mate choice hypothesis: mutations that ultimately produce a menopause phenotype can arise effectively neutrally – non-adaptively – and accumulate over time. Developing the theory provides the opportunity to introduce biological considerations beyond those used in describing the mate choice hypothesis with associated computational model (Morton et al., 2013; this section) and also an opportunity to correct some ‘misreceptions’ and misperceptions about the mate choice hypothesis (Singh and Stone, 2013; next section).

## Remarks

Combining hypotheses is common in science, and human menopause phenotype science is no exception. Researchers often describe scenarios implicitly involving multiple hypotheses, whether intentionally. The lifespan artifact hypothesis often is discussed in combination with the ‘follicular depletion hypothesis,’ which, itself, states that exhausting a viable egg supply could provide a means for a menopause phenotype to evolve: given that women live long lives, they eventually will deplete their viable egg stores. The grandmother hypothesis often is discussed in combination with the ‘senescence hypothesis,’ which, itself, states that aging could provide a means for a menopause phenotype to evolve: given that female reproductive systems senesce faster than do other, somatic systems, evolving menopause is adaptive in that non-reproductive women can continue to ensure their fitness through kin. The grandmother hypothesis interestingly also has been discussed in combination with the ‘mother hypothesis’ (e.g., Madrigal and Meléndez-Obando, 2008), which, itself, states that aging mothers increasing the survival probability for their children by avoiding risky additional pregnancies and deliveries could provide a means for a menopause phenotype to evolve (Williams, 1957; Peccei, 2001; Pavard et al., 2008). Difficulties can occur during pregnancy and childbirth in older women. Increased risk could be attributed to somatic aging rather than decreasing oocyte numbers (Cohen, 2004). From an evolutionary perspective, greatest fitness would be achieved when younger women mated, as they would be more likely to produce healthy offspring.

Myriad alternative hypotheses have been proposed to explain the human menopause phenotype: ‘reproduction-cost,’ ‘mother,’ ‘patriarch,’ ‘absent father,’ ‘reproductive conflict,’ ‘evolutionary tradeoff’ (reviewed in Morton et al., 2013, including the challenges to some, in **Table 1** therein), ‘adaptive onset’ (Harris et al., 2009), and ‘intergenerational conflict’ (Cant and Johnstone, 2008; Lahdenperä et al., 2012). Many among these undoubtedly could be included in a synthesis to formulate a theory for menopause. We chose to synthesize the trio combined herein as a minimal, parsimonious first approach. The fundamental benefit in this synthesis is that it provides an ultimate, neutral causal mechanism – mate choice – with an adaptation – extended lifespan – and an exaptation – grandmothers regaining fitness through kin selection. Narratives for the human menopause phenotype hitherto have been fraught with theoretical problems in at least one among these three components.

Other benefits derived from the synthesis and associated narrative is that they render more-plausible the processes associable with the three synthesized hypotheses. The synthesis and narrative provide bounds for the relative timing issue between the hypothesized change in mating behavior and documented increase in longevity associated with the mate choice hypothesis (considered previously, in the section Change 1: Mate-Choice Hypothesis). The 50000 years available (i.e., since the Early Upper Paleolithic) for female-specific late-onset fertility-diminishing mutations to have accumulated, allows diminished fertility and increased longevity to have coevolved. The synthesis and narrative also overcome the main theoretical problem with the lifespan artifact hypothesis. As lifespan increased, the menopause phenotype encoded by female-specific late-onset fertility-diminishing mutations would have started to express, providing an explanation why only male fertility was extended concomitantly with longevity (mentioned previously, in the section Change 2: Lifespan Artifact Hypothesis). The synthesis and narrative also overcome the main theoretical problem with the grandmother hypothesis. As older non-reproductive women emerged, they could have increased or reclaimed inclusively fitness otherwise lost, by assisting in rearing kin, providing an explanation for how the twofold-increased selective gradient might have been alleviated at least (mentioned previously, in the section Change 3: Grandmother Hypothesis).

## Prospectus

The initiating mechanism in the theory – reproduction involving only young adult females and adult males – could arise as an intrinsic consequence from genetic variation in preference and phenotype. Classic theory (Fisher, 1915, 1930, 1958; reviewed in Andersson, 1994 and Prum, 2010) suggests that the case involving male preference for younger females may be established ultimately as an arbitrary (*sensu* Prum, 2010) mate choice interaction. Assortative mating initially establishes a genetic correlation in preference and phenotype, for instance, life histories in which males prefer mating with females close in age. An equilibrium is established as a compromise between the genetic correlation and natural selection (Fisher, 1915, 1930, 1958; Lande, 1981), which operates to favor phenotypes that optimize survival (independently from phenotypes that might be preferred intersexually). This equilibrium may be affected by a neutral shift, like genetic drift or, for instance, arbitrary preference for younger mates, perturbing populations from equilibrium. Consequences for populations affected by such shifts depend on relative magnitudes between genetic correlation and genetic variation in phenotypes (Lande, 1981; Kirkpatrick, 1982): if weaker, then the equilibrium remains stable and non-equilibrium populations evolve to re-establish at different ‘positions’ within the equilibrium; if stronger, then the equilibrium becomes unstable and non-equilibrium populations evolve exponentially away from the equilibrium, a runaway sexual selection process (Kirkpatrick, 1982; Arnold, 1983; Kirkpatrick and Ryan, 1991). The word ‘arbitrary’ in this sense is intended to describe explicitly a trait that corresponds to the preference with which it coevolves, signaling only availability by its possessor to mate and to be evaluated for preference by potential mates – “an invitation to intersexual selection”; neither honest nor dishonest (because it communicates no information that could be false or falsified); neither ahistoric, stochastic, accidental, nor inexplicable (Prum, 2010). Such neutral mechanisms for preference and phenotype evolution via intersexual selection have been proposed as necessary null models for statistical analysis (Prum, 2010).

We effectively argue from such a null hypothesis perspective, initializing the theory with Lower Paleolithic human populations characterized by short lifespans and diminished late-age survival and fertility. Those populations might have established genetic correlations in preference and phenotype and, in concert with natural selection, an equilibrium. Three changes might have perturbed those populations from equilibrium, with consequences: (1) a neutral mate choice shift, a behavior change in which only young women reproduced, rendered as effectively neutral late-onset female-specific fertility-diminishing mutations, which accumulated; (2) an adaptive lifespan increase revealed the reproductive senescence phenotype encoded in the accumulated late-onset fertility-diminishing mutation genotypes that had been unexpressed in the shorter lifespan; (3) an exaptive social interaction change in which older non-reproductive women exclusively assisting in rearing grandchildren rather than giving birth to and caring for their own children produced the first grandmothers. The arbitrary mate choice shift might have involved preference for younger mates (as described in the preceding paragraph; Symons, 1979; Casterline et al., 1986; Buss, 1989; Jones, 1996; Sugiyama, 2005). The other possibility – younger females out-competing older females for access to mates (Cant and Johnstone, 2008) – constitutes an interesting adaptive alternative hypothesis for statistical analysis in future studies.

## Conclusion

The theory as we have described it herein is applied specifically to humans. If menopause is considered quantitatively on the basis of life history as a proportionately long time in adulthood during which individuals are non-reproductive (a “post-fertile life stage” *sensu* Levitis et al., 2013; Stone, 2013), then it indeed is remarkable in humans relative to other species in the animal kingdom (Alberts et al., 2013), occurring also only in killer whales (Brent et al., 2015; Franks et al., 2016) and perhaps short-finned pilot whales (Foote, 2008). We think that the ‘triumvirate hypothesis approach’ inherent in the theory might be applicable to explaining claims for menopause in other organisms, in which it is less conspicuous (Brody et al., 1923; Laws et al., 1975; Gosden et al., 1983; Kidd and Tozer, 1985; Nelson and Felicio, 1985; Marsh and Kasuya, 1986; Ottinger and Balthazart, 1986; Finch, 1990; Austad, 1993; von Saal et al., 1994; Packer et al., 1998; Holmes and Ottinger, 2003; Cohen, 2004; Goranson et al., 2005; McAuliffe and Whitehead, 2005; Minois et al., 2005; Reznick et al., 2006; Singh and Singh, 2006; Chen et al., 2007; Foote, 2008; Walker and Herndon, 2008; Atsalis and Videan, 2009; Ward et al., 2009). Alternatively, a similar synthetic approach but differing in details (e.g., hypotheses) may be required to address specific differences between humans and other organisms. Menopause in mammals, for instance, intriguingly has been associated with populations wherein female relatedness to group members increases with age (Johnstone and Cant, 2010). Such increases in relatedness can be explained on the basis of intergenerational demographics and kinship dynamics, with theoretical modeling, and have been applied to explain menopause specifically to humans (Cant and Johnstone, 2008; with supportive evidence from Finnish populations: Lahdenperä et al., 2012; with unsupportive evidence from Indonesian populations: Snopkowski et al., 2014) and killer whales (Johnstone and Cant, 2010). A framework similar to the theory could applied to elucidate menopause in killer whales, with older females functioning more as ecological information repositories than as caregivers (Brent et al., 2015; Franks et al., 2016).

The theory entails that the human menopause phenotype may be conceived as a trait with a sufficient, non-adaptive, genetic ultimate cause: mating involving only young women allowing late-onset fertility-diminishing alleles to accumulate cryptically. Increased lifespan necessarily allowed reproductive senescence to be expressed. Grandmothers may have emerged as a means to reclaim lost fitness through kin selection. Menopause thus theoretically may be considered a trait that originated neutrally, evolved adaptively, and was co-opted exaptively (Gould and Lewontin, 1979; Gould and Vrba, 1982). The theory practically could prompt research into (1) searching for and identifying real-world counterparts for the late-onset fertility-diminishing alleles in the computer simulations (described in the section Menopause), genes which then would become candidates for exerting deleterious effects on other life history traits through pleiotropy; (2) testing whether genuine grandmothering evolved prior to paternal care (as mentioned in the section Change 3: Grandmother Hypothesis); and (3) investigating whether younger females out-competing older females for access to mates could lead to a menopause phenotype (as described in the section Prospectus). Predictions formulated on the basis of the theory also could prompt empirical research. If only young adult males and adult females were to mate in a population under selection for increased longevity (e.g., Luckinbill et al., 1984), then non-reproductive adult males could emerge. This possibility could be tested by investigating whether menopausal male fruit flies could be evolved in a laboratory setting.

## Author Contributions

All authors contributed to the research described herein and the description, itself. MT reviewed and summarized data on menopause, mating behavior, life expectancy, and grandmother hypothesis descriptions. RS conceived the mate-choice hypothesis. JS formulated the synthetic theory.

## Conflict of Interest Statement

The authors declare that the research was conducted in the absence of any commercial or financial relationships that could be construed as a potential conflict of interest.
